# Development of a Measurement Procedure for Emotional States Detection Based on Single-Channel Ear-EEG: A Proof-of-Concept Study

**DOI:** 10.3390/s26020385

**Published:** 2026-01-07

**Authors:** Marco Arnesano, Pasquale Arpaia, Simone Balatti, Gloria Cosoli, Matteo De Luca, Ludovica Gargiulo, Nicola Moccaldi, Andrea Pollastro, Theodore Zanto, Antonio Forenza

**Affiliations:** 1 Department of Theoretical and Applied Sciences, eCampus University, 22060 Novedrate, Italy; marco.arnesano@uniecampus.it; 2 Department of Electrical Engineering and Information Technology, University of Naples “Federico II”, 80121 Naples, Italy; nicola.moccaldi@unina.it (N.M.); andrea.pollastro@unina.it (A.P.); 3 AWEAR Technologies Inc., San Francisco, CA 94105, USA; simone.balatti@gmail.com (S.B.); antonio@awear.us (A.F.); 4 Department of Advanced Biomedical Science, University of Naples “Federico II”, 80131 Naples, Italy; matteo.deluca@unina.it; 5 National Research Council (STIIMA-CNR), 23900 Lecco, Italy; ludovica.gargiulo@stiima.cnr.it; 6 UCSF Weill Institute for Neurosciences School, San Francisco, CA 94143, USA; theodore.zanto@ucsf.edu

**Keywords:** EEG, ear-EEG, wearable EEG, emotion recognition, single-channel, physiological measurement, signal processing

## Abstract

Real-time emotion monitoring is increasingly relevant in healthcare, automotive, and workplace applications, where adaptive systems can enhance user experience and well-being. This study investigates the feasibility of classifying emotions along the valence–arousal dimensions of the Circumplex Model of Affect using EEG signals acquired from a single mastoid channel positioned near the ear. Twenty-four participants viewed emotion-eliciting videos and self-reported their affective states using the Self-Assessment Manikin. EEG data were recorded with an OpenBCI Cyton board and both spectral and temporal features (including power in multiple frequency bands and entropy-based complexity measures) were extracted from the single ear-channel. A dual analytical framework was adopted: classical statistical analyses (ANOVA, Mann–Whitney U) and artificial neural networks combined with explainable AI methods (Gradient × Input, Integrated Gradients) were used to identify features associated with valence and arousal. Results confirmed the physiological validity of single-channel ear-EEG, and showed that absolute β- and γ-band power, spectral ratios, and entropy-based metrics consistently contributed to emotion classification. Overall, the findings demonstrate that reliable and interpretable affective information can be extracted from minimal EEG configurations, supporting their potential for wearable, real-world emotion monitoring. Nonetheless, practical considerations—such as long-term comfort, stability, and wearability of ear-EEG devices—remain important challenges and motivate future research on sustained use in naturalistic environments.

## 1. Introduction

Real-time emotion monitoring is increasingly applied in healthcare, automotive systems, occupational safety, and entertainment [1,2,3,4]. Detecting emotional states such as stress, fear, and anger enables adaptive responses and supports well-being [1]. Emotion recognition frameworks are typically based on either categorical models, which label distinct emotions, or dimensional models, such as Russell’s Circumplex Model, which maps affective states along valence and arousal axes [5,6]. Physiological signals offer objective insight into emotional processes and complement behavioral assessments [1,7]. Electroencephalography (EEG) captures cortical activity linked to affective states and is widely used in studies on anxiety, depression, and self-regulation [4,5]. Spectral features in the α, β, and γ bands are commonly analyzed, as well as the Beta-to-Alpha Ratio (BAR) [8,9,10]. Additional features such as entropy [11,12] and fractal dimension [13,14] have also demonstrated discriminative potential. Among EEG-derived features, Power Spectral Density (PSD) is frequently adopted for its balance between detection performance and computational efficiency, making it suitable for real-time applications [15]. Peripheral signals—including Electrocardiogram (ECG), Galvanic Skin Respiration (GSR), respiration rate (RR), and skin temperature (ST)—reflect autonomic activity and complement EEG in emotion classification [16,17,18].

These modalities are often processed using Support Vector Machines (SVMs), Convolutional Neural Networks (CNNs), or K-Nearest Neighbors (KNN) but recent studies have applied Convolutional Fuzzy Neural Networks (CFNNs) [19], Random Forest, and XGBoost [20] with good performances. Recent reviews emphasize the value of integrating physiological, behavioral, and subjective data to improve adaptability and context-awareness [21]. Ongoing research focuses on standardizing fusion architectures and validating them across diverse populations.

Wearable EEG technologies have enabled unobtrusive monitoring in naturalistic environments [22,23]. Ear-centered systems like cEEGrid [24,25] offer a discrete and user-friendly alternative to traditional setups. Electrodes near the mastoids (TP9, TP10) have shown sensitivity to emotional signals [24,26,27,28,29] and EEG configurations with a low number of channels are increasingly adopted for mental health tracking, sleep staging, and meditation. Signal quality, however, remains an open challenge [30]. Positioning the EEG sensor in the mastoids area could allow for minimally obtrusive acquisition while maintaining sensitivity to affective signals. Despite recent advances, reliable emotion recognition using compact, wearable EEG systems in real operating conditions is still highly demanding.

To address this gap, the present study investigates EEG signals acquired from a single-channel ear-EEG system. The adoption of a single-channel ear-EEG configuration represents a key novelty of this study. This minimally invasive setup does not require precise electrode placement or specialized personnel, enabling a plug-and-play acquisition that reduces preparation time and user discomfort. Unlike multi-electrode systems, the compact ear-centered configuration can be easily embedded into everyday wearable items (e.g., ear-cuffs), supporting un-obtrusive and continuous monitoring in real-world conditions. The present work pursues two main objectives. First, it aims to demonstrate the feasibility of electroencephalographic acquisition from a single mastoid channel by confirming the physiological rather than artifactual origin of the recorded signal. Second, it seeks to extract EEG features conveying information about emotional processes and to assess their relationships with arousal and valence levels, using both linear statistical approaches and nonlinear explainable artificial intelligence applied to artificial neural networks. This combination provides both methodological transparency and physiological interpretability, advancing the feasibility of emotion recognition using minimal EEG instrumentation.

The remainder of this paper is organized as follows. Section 2 describes the experimental and analytical framework developed for emotion recognition using single-channel ear-EEG. It introduces the hardware and software tools employed for data acquisition (Section 2.1), the experimental setup (Section 2.2.2), and the participant cohort (Section 2.2.1), followed by a detailed overview of the data analysis workflow comprising pre-processing (Section 2.3.1), feature extraction (Section 2.3.2), statistical evaluation (Section 2.3.3), and machine learning analysis (Section 2.3.4). Section 3 presents the main findings, including the outcomes of signal processing (Section 3.1), the identification of statistically (Section 3.2) and XAI (Section 3.3) significant EEG features related to arousal and valence. Finally, Section 4 discusses the neurophysiological interpretation of the results and methodological implications, while Section 5 summarizes the key contributions and outlines directions for future research.

## 2. Materials and Methods

This section outlines the experimental and analytical framework developed for emotion recognition using single-channel ear-EEG. First, an introduction of the hardware and software tools employed for EEG acquisition (Section 2.1), followed by a description of the experimental setup (Section 2.2.2) and participant cohort (Section 2.2.1) is presented. Subsequently, a detail of the data analysis workflow, designed to extract meaningful information from EEG signals and support robust classification of emotional states, is presented. In particular, the workflow consists of four main stages. EEG pre-processing is described in Section 2.3.1, covering artifact removal, signal conditioning, and strategies to handle the limitations of single-channel recordings. Section 2.3.2 presents the extraction of both spectral and non-linear features relevant to affective processing. In Section 2.3.3, the statistical analyses used to identify features that significantly differentiate emotional states across valence and arousal levels is outlined. Finally, Section 2.3.4 describes the machine learning models employed, the evaluation metrics, and the validation procedures adopted to ensure generalizable results.

### 2.1. Hardware and Software

**Figure 1:**
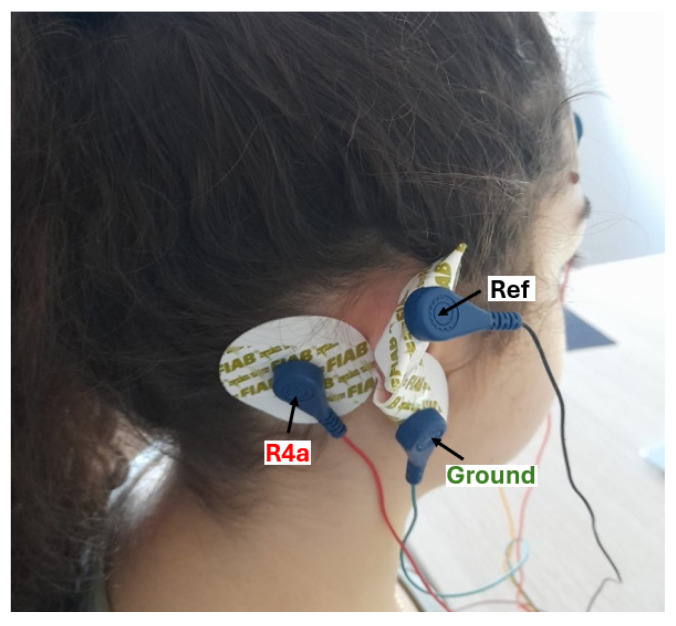
Electrodes placement: R4a (red cable), reference (black cable), and ground (green cable).

EEG data were acquired using an OpenBCI Cyton board (OpenBCI Inc., Brooklyn, NY, USA), an 8-channel (expandable to 16) 24-bit biosensing amplifier based on the ADS1299 analog front end. The system provides high-resolution measurements (0.298 μV/bit) and a signal-to-noise ratio of 121 dB. Signals were sampled at 250 Hz using standard gold-cup electrodes, and data acquisition was carried out through the OpenBCI GUI or custom acquisition software. EEG signals were recorded from four electrodes, with the analysis focusing on the R4a channel to evaluate single-channel ear-EEG performance. Electrodes were positioned at R4a (right mastoid), AF8 (right frontal), AF7 (left frontal), and L4a (left mastoid), with reference and ground electrodes on the superior and inferior regions of the right ear, respectively (Figure 1).

Participants viewed audio–visual stimuli from the OSF dataset [31], designed to evoke five emotional conditions with different different levels of arousal and valence, displayed on a 55-inch screen in a thermoigrometrically controlled environment. Emotional responses were self-reported using the Self-Assessment Manikin (SAM) scale [32], ranging from 1 to 9 for both valence and arousal.

All data processing was carried out in Python (version 3.8.16) using the Google Colab environment. EEG acquisition was performed through the OpenBCI GUI, while all subsequent preprocessing, feature extraction, and classification analyses were implemented in Python. The workflow relied on commonly used scientific libraries: NumPy and Pandas for data handling, scikit-learn for preprocessing, classical machine-learning models, and evaluation metrics, and TensorFlow/Keras for building and training the Artificial Neural Network.

### 2.2. Experimental Campaign

#### 2.2.1. Experimental Sample

A total of twenty-four healthy volunteers (18 females and 6 males, aged 23–47, mean ± standard deviation age of 29 ± 7 years) took part in the study. All participants were fully informed about the objectives of the experiment and provided written consent prior to participation. The study was conducted in accordance with the ethical principles outlined in the World Medical Association Declaration of Helsinki [33]. Approval was granted by the Ethical Committee of Psychological Research at the University of Naples Federico II (protocol number: 19/2025).

#### 2.2.2. Experimental Protocol

Participants were seated in a dark, enclosed room facing a 55-inch display, while an operator in an adjacent control room supervised video playback and EEG acquisition via OpenBCI software. To minimize movement-related artifacts, participants were instructed to remain relaxed throughout the session.

A 2-minute baseline recording was collected for each condition—Eyes Closed (EC) and Eyes Open (EO)—during which participants fixed on a white cross displayed on the screen.

Video stimuli were selected from the abovementioned OSF dataset [31] and categorized according to the Russell’s Circumplex Model of Affect: Low valence–High arousal (LV–HA; anger and fear), High valence– High arousal (HV– HA; joy and excitement), Low valence– Low arousal (LV– LA; sadness and boredom), High valence– Low arousal (HV– LA; calm and relaxation), and Neutral (indifference). Each category included at least five clips, presented in randomized order for each subject to mitigate sequence effects.

Each clip lasted approximately 2 min, resulting in a total session duration of roughly 60 min per participant, including baseline and rating periods. Immediately after viewing the videos, participants rated their emotional response using the SAM scale [32], which evaluates both valence and arousal on a 1–9 scale.

Consequently, the total dataset consisted of approximately 25 independent EEG recordings (5 videos per emotional category × 5 categories) for each of the 24 total subjects.

### 2.3. EEG-Based Emotion Detection

#### 2.3.1. EEG Data Pre-Processing

Raw EEG signals were first filtered using a zero-phase, fourth-order Butterworth band-pass filter in the range [1–45 Hz], followed by a 50 Hz notch filter to suppress power-line interference. Filtering was applied independently to each channel. The filter design was deliberately restrictive in view of the strong exposure to widespread noise sources typical of real-world applications [34,35]. Signals were then segmented into non-overlapping 1-s windows. In the neuroscience and EEG literature, these windows are commonly referred to as “epochs”. However, since the term “epoch” has a different meaning in machine learning—where it indicates a full pass through the training dataset—we opted to use the term “segments” throughout the manuscript to avoid any ambiguity. Segments exceeding 100 μV in maximum absolute amplitude (per channel) were discarded to remove high-amplitude artifacts. The remaining segments were concatenated to form the cleaned signal, and segment retention was quantified using an segment-retention index.

Signal quality was further assessed using normalized Quality Control (QC) metrics ranging from 0 to 100, where higher scores indicate better signal integrity. The following QC dimensions were evaluated:

- Event-rate scores: detection of abrupt jumps, excessive amplitude, and peak-to-peak fluctuations.
- Spectral contamination: high-frequency (HF) and low-frequency (LF) power ratios, for EMG activity and motion, respectively.
- Signal retention: percentage of clean segments.

Only sessions meeting all QC criteria (QC ≥ 60) were retained for feature extraction (see Section 2.3.2).

Following each self-report, video segments were labeled according to SAM ratings for arousal and valence separately:

- Label 0: low intensity (scores 1 to 3)
- Label 1: medium intensity (scores 4 to 6)
- Label 2: high intensity (scores 7 to 9)

To ensure balanced classes, an equal number *V* of videos were selected per label and a fixed number of segments ($E_{\min}$) was extracted from each video. This procedure guarantees comparability across participants and supports downstream classification analyses.

#### 2.3.2. EEG Features Extraction

Feature extraction was performed using 15-s sliding windows with a 2-s shift (13-s overlap). This windowing scheme was selected based on preliminary analyses showing that shorter segments yielded unstable spectral estimates, whereas windows of 15 seconds provided substantially more reliable PSD profiles with minimal additional benefit from longer durations [36,37]. The 2-s shift increased the number of available samples while preserving the temporal continuity of the emotional response. For each window, a comprehensive set of spectral, time-domain, and non-linear features was computed to characterize EEG activity.

PSD was estimated using Welch’s method with a Hamming window and an adaptive segment length defined as $n_{\mathrm{perseg}}=\min\left(N,\max\left(128,f_{s}\right)\right)$, where *N* denotes the number of samples per window (in points) and $f_{s}$ the sampling frequency (in Hz). Spectral power was computed across canonical EEG bands [38]: δ (1–4 Hz), θ (4–8 Hz), α (8–12 Hz), $\alpha_{1}$ (8–10 Hz), $\alpha_{2}$ (10–12 Hz), β (12–30 Hz), $\beta_{1}$ (12–18 Hz), $\beta_{2}$ (18–24 Hz), $\beta_{3}$ (24–30 Hz), γ (30–42 Hz), $\gamma_{1}$ (30–38 Hz), and $\gamma_{2}$ (38–42 Hz).

The Absolute Spectral Power (ASP, indicated with subscripts ‘a’) was obtained by integrating the PSD within each band, while the Relative Spectral Power (RSP, indicated with subscripts ‘r’) was computed by normalizing ASP values to the total power across all bands, thereby reducing inter-subject variability. Two additional spectral ratios were derived to capture cross-band dynamics: (i) Alpha–Beta Ratio (ABR) and (ii) Alpha–Gamma Ratio (AGR), each calculated as: $20\cdot \log_{10}\left(0.6\cdot \beta \left(or\alpha \right)/\gamma \right)$. The constant factor 0.6 was empirically introduced as a regularization term to stabilize ratio estimates in low-amplitude windows. The Total Spectral Power was also computed as the sum of ASP values across all bands.

To characterize the morphological and dynamical properties of the EEG signal, the following descriptors were extracted from each window:

- Hjorth parameters: Activity (signal variance), Mobility (square root of the variance ratio between the first derivative and the signal), and Complexity (ratio between the mobility of the signal and that of its derivative), describing amplitude dynamics and spectral composition [39].
- Zero-Crossing Rate (TD_ZCR): number of sign changes per second, indicative of oscillatory richness and cortical activation [40].
- Fractal Dimensions: Petrosian (TD_PFD) and Higuchi (TD_HFD), estimating signal complexity based on direction changes and multi-scale irregularity [41].
- Lempel–Ziv Complexity (TD_LZC): quantifies the diversity of binary patterns after median-based binarization, reflecting temporal variability and unpredictability [42].
- Permutation Entropy (TD_PermEn): computed with embedding order = 3 and delay = 1, representing the diversity of ordinal patterns; calculated on z-scored signals to ensure amplitude invariance [43].
- Sample Entropy (TD_SampEn): calculated with m=2 and r=0.2·std on z-scored windows, measuring signal irregularity and unpredictability [44].
- Statistical Descriptors: Coefficient of Variation (CV), Skewness, and Kurtosis, characterizing dispersion, asymmetry, and peakedness of the amplitude distribution [45].

In the end, a total of 39 features (for details see the Table A1 in the Appendix A), were considered for further analysis. All computations incorporated numerical safeguards to handle low-variance windows, missing values, and potential numerical instabilities. In particular, divisions by extremely small values were avoided by applying lower bounds to denominators in ratio-based features, and the propagation of undefined or non-finite values (e.g., NaN or Inf) through the processing pipeline was prevented. These measures ensured robust and reproducible feature estimation across all sessions.

#### 2.3.3. Statistical Analysis

Statistical analysis was conducted to evaluate condition-related differences in EEG-derived features across valence and arousal. All tests were two-sided with a significance level of α=0.05, and implemented in Python 3.8.16 using the SciPy, Pingouin, and statsmodels libraries. For three-group comparisons (i.e., low, medium, and high levels of valence or arousal), a non-parametric Kruskal–Wallis omnibus test was applied. When significant, post-hoc pairwise contrasts were evaluated using two-sided Mann–Whitney U tests, with multiple-comparison correction via the Benjamini–Hochberg False Discovery Rate (FDR_BH_). Effect sizes for pairwise contrasts were reported as Cliff’s delta (δ) or rank-biserial correlation, and interpreted according to conventional thresholds: |δ|≥0.474 (large), 0.33≤|δ|<0.474 (medium), 0.147≤|δ|<0.33 (small). For binary comparisons, Welch’s t-test with Cohen’s *d* was used when both normality (Shapiro–Wilk test) and homoscedasticity (Levene’s test) assumptions were satisfied per feature. Otherwise, the Mann–Whitney U test with Cliff’s delta was preferred. Cohen’s *d* values were interpreted using standard thresholds [46]: |d|≥0.8 (large), 0.5≤|d|<0.8 (medium), 0.2≤|d|<0.5 (small).

To identify robust and interpretable features, a multi-step screening procedure was applied. Only features showing statistically significant and large effects were retained if (i) at least 30% of total subjects exhibited the effect and (ii) at least 50% of those subjects showed the same direction of change (within-subject consistency). Surviving features were ranked per comparison using a composite score that combines statistical significance, effect magnitude, and cross-subject consistency:

(1) $$\mathrm{score}=-\log_{10}\left(median\left(p\right)\right)\times \mathrm{normalized}\;\mathrm{effect}\;\mathrm{size}\times \mathrm{consistency}$$

The *p*-value contribution was capped prior to logarithm calculation so that extremely small *p*-values do not strongly impact the combined metric. The median *p*-value was bottom-capped at 1e-12 to keep the score driven by all three components. The results of this analytical framework, including model performance and feature relevance distributions, are presented in Section 3.2.

#### 2.3.4. Machine Learning Analysis

All models were trained and tested on the same balanced dataset containing two emotion levels (low vs. high) for both arousal and valence, ensuring comparability and preventing class bias. EEG data were structured into matrices $X\in R^{R\times K}$, where $R=N\cdot C\cdot V\cdot E_{\min}$ represents the total number of segments (*N* subjects, *C* classes, *V* videos per class, and $E_{\min}$ the global minimum number of usable segments per video across all subjects), and K=39 denotes the number of features extracted per segment. The target variable *y* encodes class labels.

The primary classification task was performed using a feedforward fully connected Artificial Neural Network (ANN). The choice of employing ANN is motivated by the primary objective of this study, which focuses on identifying informative EEG features rather than maximizing classification accuracy. Compared to statistical approaches relying on linear assumptions, ANNs provide a richer and more flexible function space due to the hierarchical composition of nonlinear transformations. This structure enables the modeling of both global nonlinearities and fine-grained local variations in the relationship between EEG features and emotional states, which are typical of biological signals. Traditional classifiers such as SVMs, decision trees, or linear models operate within more rigid hypothesis spaces or rely on static kernels, limiting their ability to capture deep nonlinear interactions. To ensure a stable estimation of model parameters with the available dataset, the classification task was reformulated as a binary problem (low vs. high). Multi-class settings require more complex decision boundaries and substantially larger datasets, whereas the binary formulation provides a more appropriate balance between sample size, model complexity, and the intrinsic variability of EEG-based affective responses [47]. Network architecture was optimized through a grid search over multiple hyperparameters, including: (i) number of hidden layers L∈{1,2,3}, (ii) number of nodes per layer selected from {16, 32, 64, 128} with layer-wise combinations, (iii) activation function (ReLU or tanh), (iv) batch size ∈{128, 256}, and (v) weight regularization factor $\lambda \in \{0,5\times 10^{-4}\}$. Fourteen distinct node arrangements were combined with eight hyperparameter configurations, resulting in a total of 112 network configurations explored as part of a structured hyperparameter search. The explored ranges were defined during preliminary analyses to remain compatible with the available sample size while still allowing the investigation of different network depths, widths, and activation functions. This systematic exploration was not intended to optimize predictive performance but rather to assess the stability of feature relevance across a diverse set of nonlinear models.

Model training was performed independently for each subject, following an intra-subject analysis design. Given the exploratory and proof-of-concept nature of this study, the modeling strategy was intentionally based on an intra-subject design. This choice is justified by the limited sample size and by the substantial inter-individual variability typically observed in EEG-based affective responses [48]. Training models separately for each participant reduces the confounding effects of cross-subject heterogeneity and allows the extraction of subject-specific patterns that would otherwise be diluted in group-level analyses. As a consequence, statistical power is primarily defined at the intra-subject level, which is appropriate for feasibility studies aimed at validating the physiological plausibility of single-channel ear-EEG rather than achieving population-level generalization. Since the objective of this analysis was not to maximize predictive performances but rather to identify the most informative EEG features, the focus was placed on the interpretability of model predictions through XAI techniques. For every participant, the dataset was divided into 60% for training, 20% for validation, and 20% for testing. A stratified 10-fold cross-validation was applied ensuring that each fold preserved the original class distribution. This procedure allowed all samples to be used both for training, validation and test across folds, providing a more reliable estimate of model performance and reducing the risk of overfitting, particularly important given the limited size of the dataset.

The network was trained using the Adam optimizer (initial learning rate = $1\times 10^{-3}$) and the categorical cross-entropy loss function. Early stopping was applied after 20 consecutive segments without improvement in validation loss. Mean accuracy and loss metrics were computed per subject and reported separately for training, validation, and test sets.

For each subject, the optimal ANN configuration was selected as the model achieving the highest test accuracy among all trained architectures. Feature relevance was then estimated using the Gradient × Input and Integrated Gradient methods, computed exclusively on the training set that subsequently yielded the highest test accuracy, ensuring that relevance scores were derived from a model trained under conditions leading to robust generalization (i.e., training accuracy never below 90%).

Gradient × Input is a gradient-based saliency approach originally proposed by Simonyan et al. [49] and further analyzed in comparison with DeepLIFT [50] and LRP [51]. For each feature $x_{i}$, relevance was computed as the absolute value of the product between the feature and the gradient of the class output with respect to that feature:

(2) $$R_{i}=\left|x_{i}\cdot \frac{\partial y_{c}}{\partial x_{i}}\right|,$$

where $R_{i}$ denotes the relevance of feature *i* for class *c*, $x_{i}$ is the feature value, and $y_{c}$ is the predicted output score for class *c*.

A complementary gradient-based saliency technique, Integrated Gradients (IG) [52], was employed to further assess the robustness of feature relevance estimation. IG quantifies the contribution of each input feature to the model prediction by integrating the gradients of the output with respect to the input along a straight interpolation path between a baseline reference and the actual input. Given a neural network model F(x), an input vector x, and a baseline $x^{\prime }$, the attribution associated with the *i*-th feature is defined as:

(3) $${\mathrm{IG}}_{i}\left(x\right)=\left(x_{i}-x_{i}^{\prime }\right)\int_{\alpha =0}^{1}\frac{\partial F\left(x^{\prime }+\alpha \left(x-x^{\prime }\right)\right)}{\partial x_{i}}\;d\alpha .$$

This formulation accumulates gradient information along the interpolation path, mitigating issues such as gradient saturation and yielding more stable and reliable attributions compared to standard gradient-based methods. For both methods, the absolute value was used to aggregate both positive and negative contributions. Relevance scores were averaged across all segments and subsequently normalized to the range [0, 1] per subject. Features with normalized relevance scores greater than 0.5 in both classes were retained for each subject. To ensure sufficient feature representation, a minimum of eight features per subject was enforced; when fewer than eight features exceeded the threshold, the top-ranked features closest to the cutoff were included. Subsequently, only features that were deemed relevant in at least 30% of subjects (i.e., in five or more out of sixteen) were retained for group-level analyses. For each such feature, cumulative relevance scores were computed by summing contributions across subjects in which the feature was significant.

For comparative purposes, three traditional classifiers were considered: (i) Support Vector Machine (SVM) with RBF kernel, (ii) *k*-Nearest Neighbors (kNN) with k=5, and (iii) Logistic Regression (LR) with a maximum of 1000 iterations. All models were trained and tested on the same balanced dataset containing two emotion levels (low vs. high) to ensure comparability and prevent bias. Mean classification accuracies were computed as the average of per-subject test accuracies and compared against those obtained from the ANN.

The results of this analytical framework, including model performance and feature relevance distributions, are presented in Section 3.3.

## 3. Results

This section presents the results obtained from the analytical framework described in Section 2. Section 3.1 reports the outcomes of the EEG pre-processing and feature extraction pipeline, including signal conditioning, artifact rejection, and the balancing of segments and video samples across classes. These steps ensure that subsequent analyses are performed on high-quality, well-structured data. In Section 3.2, we present the results of statistical analyses conducted to identify features that reliably differentiate emotional states across valence and arousal levels.

Finally, Section 3.3 reports the classification performance of machine learning models trained with the extracted features, illustrating both accuracy and feature relevance.

### 3.1. EEG Pre-Processing and Feature Extraction

**Figure 2:**
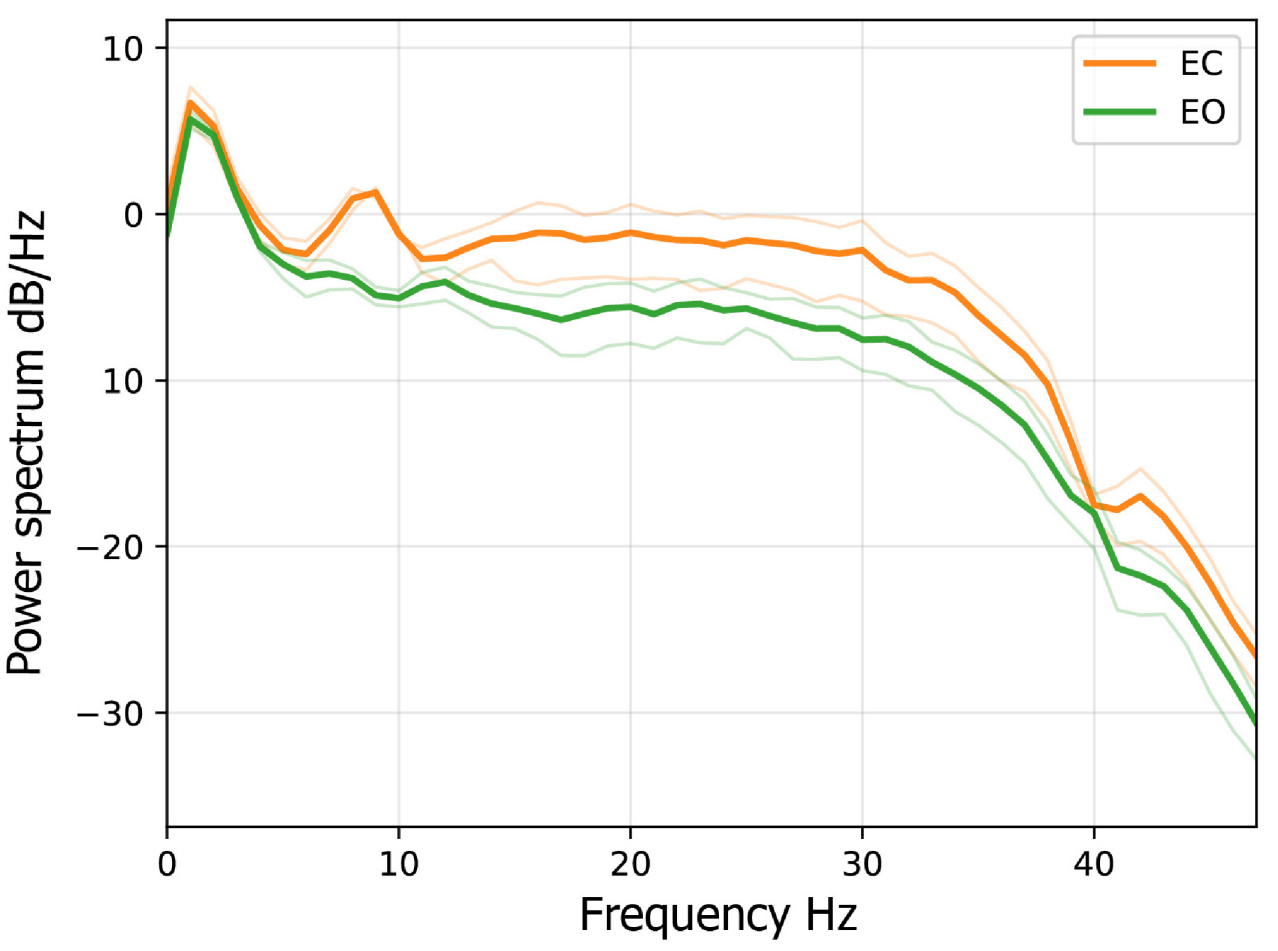
Example of spectra comparison between open (EO) and closed eyes (EC) sessions performed on subject 12.

**Figure 3:**
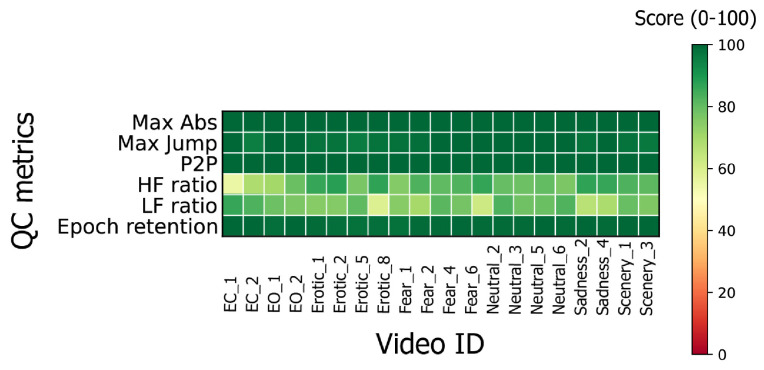
Example of QC analysis performed on subject s12 presenting the heatmap of normalized QC metric scores for all videos.

Following the pre-processing steps described in Section 2.3.1, a fixed amplitude threshold of 100 μV was applied to each recording. This procedure resulted in a variable number of discarded segments per recording, with segment retention ranging from 0% to 100% (mean = 90.24%, SD = 15.62%). In addition, 120 video segments (approximately 20% of the total) were excluded because they did not meet the minimum Quality Control (QC) threshold. For each subject, the physiological validity of the EEG signal was verified by comparing the power spectra recorded during the eyes-open and eyes-closed sessions, as shown in Figure 2. An example of the QC report is shown in Figure 3. The summary of QC scores achieved by each session is shown in Table A2 in the Appendix A.

Specifically, the reasons for exclusion were: (i) 5 videos were rejected due to abrupt jumps, excessive amplitude, or peak-to-peak fluctuations (event-rate scores); (ii) 92 videos exhibited spectral contamination, including high-frequency (HF, n=79), and low-frequency (LF, n=13) anomalies; (iii) 23 videos were excluded due to combined violations of both event-rate and spectral QC metrics. All counts are mutually exclusive and sum to the total number of excluded videos. Two out of 24 participants were removed from further analysis due to missing class labels based on their self-reported data. Given the unbalancing of class compositions due to the subjective ratings and the exclusion of approximately 20% of video stimuli due to QC issues, the number of videos held per subject was 4 videos per class and 43 segments per video.

EEG feature extraction, as described in Section 2.3.2, resulted in a total of 39 features, including 27 spectral features capturing ASP and RSP, cross-band ratios, and total power, 9 non-linear features quantifying signal complexity and dynamics (e.g., Hjorth parameters, entropies, fractal measures, and zero-crossing rate), and 3 statistical features describing the amplitude distribution (coefficient of variation, skewness, and kurtosis).

### 3.2. Statistical-Based Analysis

Table 1 summarizes the EEG features that showed statistically significant differences (*p* < 0.05 and at least large effect size given by Cliff’s |δ|≥0.474) in the intra-subject analysis. For each participant, the table reports the features that discriminate between the pair of conditions within both the arousal and the valence dimensions (Low_vs_High, Low_vs_Mid, and Mid_vs_High). These results highlight the inter-individual variability in the neural correlation of emotional processing as well as the specific frequency bands and spatial patterns most sensitive to changes in emotion state.

Across subjects, a subset of spectral features—particularly those related to high-frequency oscillations (β and γ bands)—emerged as significant. These features, often in their absolute form (e.g., $\beta_{a}$, $\gamma_{a}$), suggest that intra-subject differences in cortical activation intensity are linked to emotional state change. In 9 subjects, both arousal and valence comparisons (low–mid, mid–high, low–high) revealed concurrent activation patterns in β and γ bands, indicating that high frequencies may capture both the intensity and valence of emotional processing. Despite this general trend, a considerable degree of inter-individual variability was observed. Some participants showed strong effects mostly in arousal contrasts, while others exhibited significant differences primarily in valence. This variability underlines the subject-specific nature of EEG-based emotional signatures and supports the fact that emotion-related neural responses are casted by individual factors. Time-domain and nonlinear features (e.g., Hjorth parameters, TD_HFD, TD_LZC, and TD_PermEn) appeared less frequently but were significant in a subset of participants, suggesting that temporal complexity measures may capture unique aspects of individual emotional regulation dynamics that complement the frequency-domain results. Overall, intra-subject results emphasize that while group-level trends indicate dominant roles for β and γ bands, the combination of spectral and temporal descriptors provides a richer and individualized characterization of emotional states. These findings suggest that personalized modeling approaches could be more accurate in the field of emotional recognition via single-channel EEG signals, where intra-subject variability should be treated not as noise but as an informative marker of individual emotional processing strategies.

**Figure 4:**
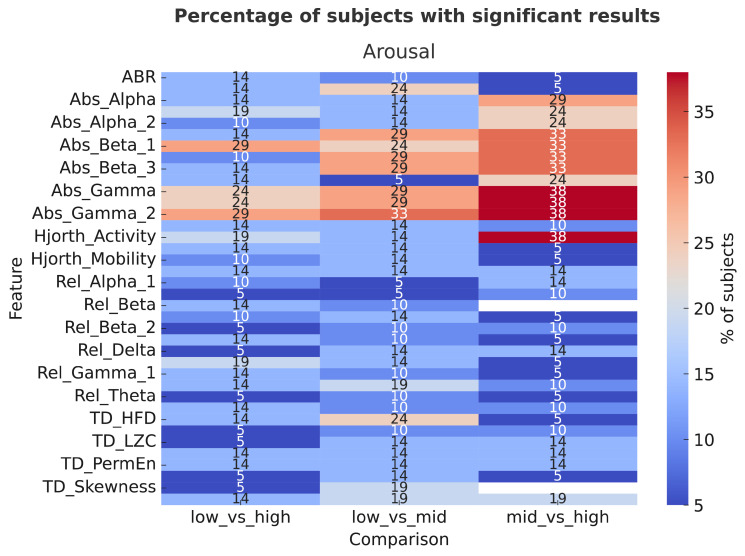
Heatmap of inter-subject analysis for arousal comparison, showing the percentage of subjects (%) that presented significant and large effects for each EEG feature (y axis) and comparison (x axis).

**Figure 5:**
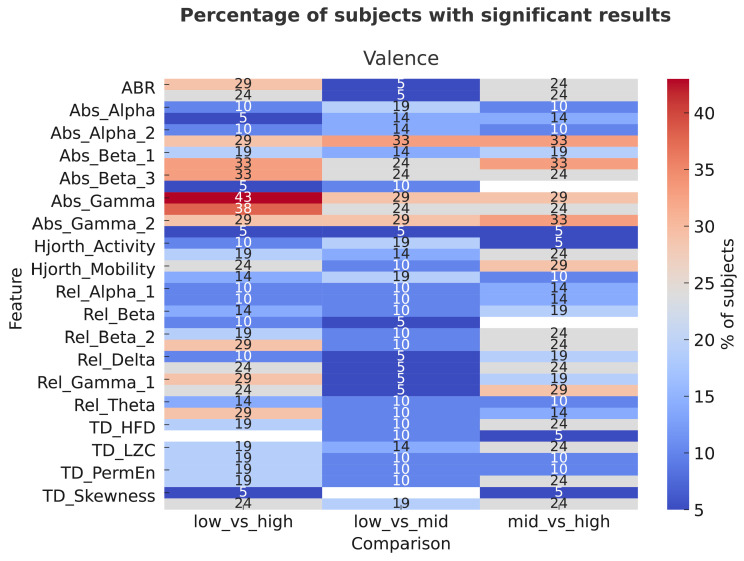
Heatmap of inter-subject analysis for valence comparison, showing the percentage of subjects (%) that presented significant and large effects for each EEG feature (y axis) and comparison (x axis).

In addition, inter-subject analysis was performed to assess the proportion of subjects that exhibit statistically significant effects (*p* < 0.05) with at least a large effect size. Separate analyses were conducted for the two affective dimensions and results are shown in Figure 4 and Figure 5.

Concerning the arousal, the most prominent results were observed for absolute spectral power features, particularly $\gamma_{a}$, $\gamma_{2_{a}}$, $\beta_{1_{a}}$, and $\beta_{3_{a}}$. These features showed significant and large effects in approximately 30–38% of participants, especially in the mid–high and low–high comparisons. This indicates that changes in high-frequency EEG activity (β and γ bands) are more sensitive to differences between medium and high arousal levels than between low and medium levels. Moderate proportions (about 20–25%) also exhibited large effects for Hjorth Activity, particularly in the mid–high comparison, suggesting a modest contribution of time-domain dynamics to arousal discrimination. In contrast, relative power features ($\alpha_{r}$, $\beta_{r}$, $\gamma_{r}$) and nonlinear temporal descriptors (TD_HFD, TD_LZC, TD_PermEn) exhibited smaller effects (<15%), indicating lower sensitivity to arousal variations. For valence, the largest proportion of significant effects (up to 40–43%) was again found in absolute spectral power features, especially $\gamma_{a}$ and $\gamma_{2_{a}}$, primarily in the low–mid and low–high comparisons. This suggests that γ-band power plays a central role in differentiating affective valence levels, particularly between neutral and positive emotional states. Other features, such as $\beta_{1_{a}}$ and $\beta_{3_{a}}$ showed moderately high proportions (30–33%) of significant large effects, while relative spectral power and temporal dynamics contributed less (<20%) across comparisons. Overall, absolute amplitude measures in the high-frequency EEG bands (β and γ) emerged as the most reliable indicators for both arousal and valence, showing consistent large effects in a substantial fraction of participants. Arousal effects were more pronounced in the mid–high and low–high contrasts, suggesting that neural differentiation strengthens with emotional intensity. On the other hand, valence effects peaked in the low–mid comparison, possibly reflecting greater neural differentiation between unpleasant and neutral states than between neutral and pleasant ones.

The results from feature screening analysis, conducted on features that were both statistically significant and exhibited at least a large effect size, are shown in Table 2.

Within the arousal dimension, features such as $\beta_{a}$, $\beta_{1_{a}}$, $\gamma_{a}$, and $\gamma_{2_{a}}$ achieved the highest scores, reflecting both low median *p*-values and large normalized effect sizes. These features demonstrated strong internal coherence, with consistency values exceeding 0.75 in the mid–high comparison, suggesting that higher arousal states are characterized by enhanced beta and gamma power across subjects. The robustness of these effects indicates that high-frequency activity reliably differentiates levels of arousal and could serve as a stable neural marker of physiological activation intensity. A similar result was found for the valence dimension, where absolute γ- and β-related features dominated the top ranks, even if with slightly lower consistency values. This pattern are aligned with previous evidence suggesting that γ-band power is particularly sensitive to valence, capturing distinctions between neutral and emotionally charged stimuli. The screening confirms that absolute high-frequency spectral power (β and γ bands) represents the most reliable and generalizable EEG signature of emotional states, while intra-individual consistency underscores their potential for robust modeling in affective computing applications. Finally, by integrating the intra- and inter-subject analyses, the statistical results suggest that absolute spectral power features, measured in the mastoid region, can serve as common predictors for general modeling of emotional state responses. However, to achieve robust and individualized predictions, intra-subject variability should be incorporated to fine-tune the model according to each participant’s specific profile.

### 3.3. Machine Learning-Based Analysis

The exploratory machine learning-based analysis on the same dataset of the statistical-based one produced not significant results. In particular, no models overcame the threshold of 40% accuracy on a three-class classification problem, both for arousal and valence dimensions. Cross-subject ML classification confirmed the importance of individualized modeling: training on multiple participants and testing on unseen subjects resulted in lower performance, reflecting the intrinsic variability in feature relevance profiles across individuals. Therefore, a further data reduction procedure was carried out and a total of six subjects were excluded from the analysis. Four subjects were excluded based on unreliable self-reported ratings, defined as deviations of at least three points from the group mean for the same stimuli in more than 30% of the videos. Finally, two participants were identified as outliers based on their EEG feature distributions, exhibiting mean feature values exceeding two standard deviations from the group mean in more than 30% of the total features. The remaining participants were retained for all subsequent analyses. For each subject, the ANN was trained and evaluated across all 112 network configurations, and the optimal model was selected based on maximum test accuracy (best models for each subject are reported in Table A3 and Table A4 for arousal and valence, respectively in the Appendix A). Using these best-performing models, gradient-based relevance scores for Gradient × Input and IG were computed for all 39 EEG features on the training set. Features with normalized relevance scores exceeding 0.5 in both classes were retained per subject, with a minimum of eight features enforced. At the group level, only features identified as relevant in at least 30% of subjects (i.e., ≥5/16) were considered for further analysis. These features are reported in Table 3 and Table 4 (for valence and arousal, respectively) concerning the Gradient × Input method and in Table 5 and Table 6 concerning the IG method.

For valence classification, sixteen features were identified in at least 30% of subjects using the Gradient × Input analysis. TD_SampEn and TD_LZC were the most consistently selected features (68.75% of subjects), followed by $\beta_{2_{r}}$ (62.5%), $\alpha_{2_{a}}$ (56.25%), $\gamma_{2_{r}}$ (56.25%), TD_HFD (43.75%), $\beta_{3_{r}}$ (43.75%), Hjorth_Mobility (43.75%), Hjorth_Complexity (37.5%), $\theta_{r}$ (37.5%), TD_ZCR (37.5%), $\beta_{3_{a}}$ (31.25%), $\alpha_{2_{r}}$ (31.25%), $\beta_{1_{a}}$ (31.25%), TD_PFD (31.25%), and TD_PermEn (31.25%). Using the IG method, twelve features met the same selection criterion. The most frequently retained feature was $\theta_{r}$ (62.5% of subjects), followed by TD_HFD (50%), TD_SampEn (43.75%), TD_LZC (43.75%), $\beta_{3_{a}}$ (37.5%), $\beta_{1_{r}}$ (37.5%), TD_PFD (37.5%), TD_PermEn (37.5%), Hjorth_Complexity (31.25%), $\delta_{a}$ (31.25%), $\beta_{2_{a}}$ (31.25%), and ABR (31.25%). Cumulative relevance scores, summed across subjects in which each feature was identified as relevant, indicated that TD_LZC, TD_SampEn, $\beta_{2_{r}}$, $\alpha_{2_{a}}$, and $\gamma_{2_{r}}$ contributed most strongly to valence predictions, consistently showing the highest aggregate relevance across both attribution methods. For arousal classification, the Gradient × Input analysis retained twenty-three features in at least 30% of subjects. TD_LZC (68.75%), TD_HFD (62.5%), TD_SampEn (62.5%), and $\gamma_{2_{r}}$ (62.5%) were most consistently selected, followed by $\gamma_{2_{a}}$ (56.25%), $\theta_{r}$ (50%), $\alpha_{2_{r}}$ (50%), $\beta_{2_{r}}$ (43.75%), Hjorth_Complexity (43.75%), TD_PFD (43.75%), TD_PermEn (43.75%), $\beta_{2_{a}}$ (37.5%), $\gamma_{1_{a}}$ (37.5%), $\gamma_{1_{r}}$ (37.5%), TD_Kurtosis (37.5%), $\theta_{a}$ (37.5%), $\delta_{a}$ (31.25%), $\beta_{3_{a}}$ (31.25%), $\alpha_{1_{r}}$ (31.25%), $\beta_{1_{r}}$ (31.25%), AGR (31.25%), and ABR (31.25%). The IG analysis yielded twenty-four features meeting the same threshold. TD_SampEn (68.75%) and TD_LZC (62.5%) were the most frequently selected features, followed by $\gamma_{2_{a}}$ (56.25%), $\beta_{1_{a}}$ (56.25%), $\beta_{3_{r}}$ (56.25%), $\gamma_{a}$ (50%), $\gamma_{1_{a}}$ (50%), $\theta_{a}$ (50%), $\delta_{r}$ (43.75%), $\gamma_{2_{r}}$ (43.75%), TD_PFD (43.75%), TD_PermEn (43.75%), $\gamma_{r}$ (37.5%), $\beta_{2_{r}}$ (37.5%), $\alpha_{1_{r}}$ (37.5%), Hjorth_Complexity (37.5%), TD_ZCR (37.5%), $\beta_{3_{a}}$ (37.5%), $\alpha_{1_{a}}$ (37.5%), $\beta_{a}$ (37.5%), TD_HFD (31.25%), $\theta_{r}$ (31.25%), AGR (31.25%), and ABR (31.25%). Cumulative relevance scores confirmed that TD_LZC, TD_HFD, TD_SampEn, and $\gamma_{2_{r}}$ dominated the contribution to arousal predictions, highlighting the central role of non-linear complexity measures and high-frequency spectral components in modeling arousal-related EEG dynamics. Across subjects, test accuracies for models trained on all 39 features ranged from 50.82% to 95.90% (mean accuracy = 70.25%, STD = 12.37%) for valence and 56.63% to 80.93% (mean = 71.80%, STD = 6.86%) for arousal.

**Figure 6:**
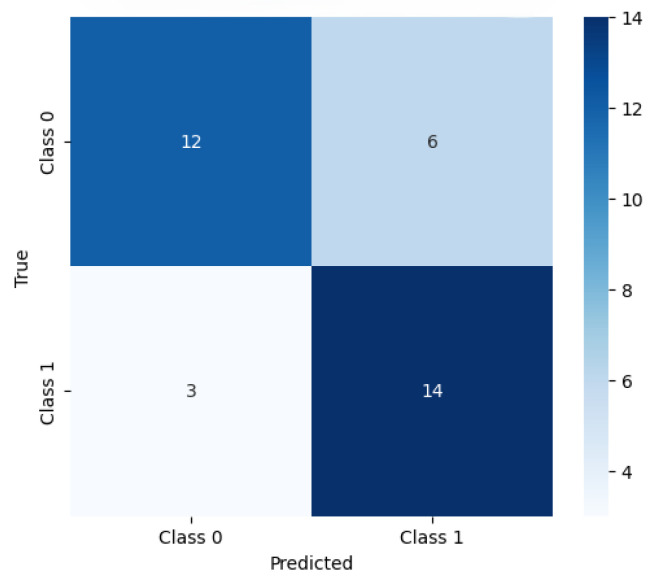
Typical confusion matrix of the ANN classifier computed on the test set. The matrix summarizes the distribution of predictions across the two emotion classes (0—Low and 1—High), highlighting which classes are most frequently confused.

To complement these subject-level accuracy results, an additional representative confusion matrix is shown in Figure 6, illustrating the distribution of predictions across the two emotion classes and highlighting the most common misclassification patterns. This visualization provides a clearer view of class-specific behavior of the ANN model, complementing the precision, recall, and F1-scores reported in Table A5 and Table A6.

## 4. Discussion

This study had two primary goals. First, to demonstrate the feasibility of using a single mastoid EEG channel, ensuring that the recorded signals reflect genuine physiological activity rather than artifacts. Second, to identify EEG features informative of emotional states and evaluate their association with arousal and valence using both classical statistical methods and explainable AI applied to artificial neural networks.

Minimal-channel and ear-EEG recordings have been increasingly explored as alternatives to conventional multi-channel scalp EEG, particularly in the context of wearable and unobtrusive affective computing systems [53,54]. Multi-channel EEG studies on emotion recognition typically report higher classification accuracies, benefiting from spatial information distributed over frontal, temporal, and parietal regions. However, such setups rely on dense electrode montages, gel-based contacts, and controlled laboratory conditions, which limit their applicability in daily-life and long-term monitoring scenarios. In contrast, minimal-channel and ear-EEG approaches prioritize usability, comfort, and ecological validity, at the cost of reduced spatial resolution and increased inter-subject variability.

Regarding the first objective, results demonstrate that the mastoid channel captured meaningful EEG activity rather than noise or muscular artifacts. Following the pre-processing steps described in Section 2.3.1, only 120 video segments ( 20% of the total) were excluded due to failing QC criteria, including abrupt amplitude jumps, spectral contamination, or combined violations of event-rate and spectral metrics. Moreover, the consistent detection of emotion-related EEG components across participants supports the physiological validity of the signal, demonstrating that this minimal setup is indeed effective and provides reliable information on cortical dynamics. This finding is particularly relevant for wearable and unobtrusive emotion-monitoring systems for daily-life applications, where reducing the number of electrodes improves comfort and usability, thus favoring a rapid spreading of the technology.

These findings are in line with prior ear-EEG and behind-the-ear EEG studies, which have shown that temporo-mastoid placements can reliably capture cortical dynamics related to cognitive and affective processing, albeit with lower signal amplitude and higher sensitivity to individual anatomy compared to scalp EEG [55,56].

Compared to multi-channel scalp EEG, ear-EEG and single-channel mastoid recordings typically yield lower absolute signal-to-noise ratios and reduced spatial specificity. Nevertheless, previous work has shown that affective information is often encoded in spectral and temporal features that do not strictly depend on spatial topography. The present findings support this view, demonstrating that emotionally relevant information can be extracted even from a single-channel setup, provided that appropriate preprocessing, feature selection, and validation strategies are employed [54,57].

Concerning the second objective, statistical and ML analysis revealed a subset of EEG features significantly associated with both arousal and valence dimensions.

Inter-subject statistical analyses identified (i) spectral ratios (ABR and AGR), (ii) high-frequency ABS (β- and γ-band activity), and (iii) entropy-based complexity metrics (TD_SampEn and TD_PermEn) as the most informative features with large effect sizes and consistent patterns across participants. Further analysis revealed that γ power is particularly sensitive to valence differences (especially low–mid contrasts), while β power reliably differentiate higher arousal levels (mid–high and low–high comparisons). These results are broadly consistent with multi-channel EEG emotion-recognition studies, which frequently report increased β and γ activity during emotionally salient and high-arousal conditions, as well as modulation of entropy and complexity measures reflecting changes in cortical engagement and information processing [58,59,60]. Intra-subject statistical analyses confirmed these patterns: absolute β- and γ-band power consistently achieve is the highest scores with strong effect sizes and internal consistency, whereas time-domain and nonlinear features, such as Hjorth parameters and TD_PermEn, are statistically significant only in a few subsets of participants.

At the individual level, this observation aligns with previous affective EEG literature, where inter-individual differences in emotional processing, baseline rhythms, and cognitive strategies are known to strongly influence feature relevance. Such variability is typically attenuated in multi-channel scalp EEG by spatial averaging, but becomes more prominent in minimal-channel recordings [54,61].

Regarding ML analysis, the proposed ANN–XAI framework achieved higher classification accuracies than conventional models while providing interpretable measures of feature relevance. Using the IG-based attributions and Gradient×Input method, the most salient EEG features for arousal and valence discrimination were quantitatively identified. In particular, TD_LZC, TD_SampEn, and $\beta_{2_{r}}$ contributed most strongly to valence prediction while TD_HFD, TD_LZC, TD_SampEn dominated the contribution to arousal prediction. These results are in line with previous literature. Specifically, non-linear complexity measures, such as Lempel–Ziv complexity and entropy-based metrics, quantify the temporal irregularity and dynamics of EEG signals and are well-suited to characterize the evolution of spatiotemporal activity patterns in non-linear, high-dimensional systems such as the brain [62]. From a neurophysiological perspective, emotional states are not localized phenomena but emerge from the interaction of distributed cortical and subcortical networks, leading to changes in the variability of neural activity. Consequently, complexity measures can provide informative markers of changes in underlying emotional states, both in terms of arousal and valence [63]. Moreover, $\beta_{2_{r}}$ emerged in valence level classification. In the literature, high-beta oscillations have been linked to reward-related and positive outcome processing [64]. In particular, oscillatory activity in the high-beta range has been proposed to mediate the synchronization of distributed brain regions involved in learning from positive outcomes and in motivating adaptive behavior [65].

The machine learning results further clarify this distinction. Cross-subject ML classification confirmed the importance of individualized modeling: training on multiple participants and testing on unseen subjects resulted in lower performance, reflecting the intrinsic variability in feature relevance profiles across individuals. This outcome is consistent with prior ear-EEG and wearable EEG studies, which often report reduced inter-subject generalization compared to within-subject models [54,55].

Importantly, this apparent discrepancy between statistically significant group-level effects and limited inter-subject predictive performance reflects the fundamental distinction between explanation and prediction. Statistical tests are designed to detect consistent effects at the group level, even when such effects are weak and embedded in high inter-individual variability. In contrast, subject-independent predictive models require discriminative patterns to be sufficiently stable and geometrically aligned across individuals—a condition that is particularly challenging to meet in minimal-channel EEG recordings and small to moderate-sized datasets [54,57].

The overlap between statistical significance and XAI-derived relevance reinforces the robustness of high-frequency spectral markers and entropy measures as indicators of emotional state. Both approaches converge on the conclusion that absolute spectral power in β and γ bands, along with signal complexity metrics, reliably encode emotional intensity and valence. Temporal dynamics captured by entropy and Hjorth features complement these spectral signatures by reflecting individualized aspects of emotional processing. The recurrence of high-frequency activity was particularly noteworthy. Absolute β power reflect affective engagement and approach-related valence, whereas γ power was associated with emotional salience and perceptual integration, especially under high arousal or attentional focus [58,59,60,66,67]. Increased α power indicate lower cortical activation and reduced arousal [8,68]. Entropy measures complemented these findings: higher TD_SampEn and TD_PermEn values reflect increased signal irregularity and reduced temporal predictability, with sample entropy discriminating high-arousal states [69] and permutation entropy capturing both valence and arousal differences [70].

From the perspective of wearable neurotechnology, these findings highlight both the promise and the current limitations of single-channel mastoid EEG for affective computing. The demonstrated ability to capture physiologically meaningful and interpretable emotional markers supports the feasibility of highly unobtrusive emotion-monitoring systems. At the same time, the limited inter-subject predictability underscores the need for larger datasets, improved normalization strategies, or hybrid modeling approaches to enhance generalization [53,54]. Additionally, different models can be considered for emotion recongition, such as STRFLNet and FMLAN [71,72].

In summary, the present work contributes novel evidence that a single mastoid EEG channel can encode emotionally relevant information consistent with established affective EEG markers, while explicitly characterizing the trade-offs between interpretability, generalizability, and wearability. These results position the proposed approach as a methodologically sound proof-of-concept within the broader landscape of minimal-channel EEG and wearable affective neurotechnology. Future studies will focus on validating solutions optimized from an ergonomic perspective as well, in order to confirm their feasibility under conditions where the sensing apparatus is minimized in size.

## 5. Conclusions

This study demonstrates the feasibility and physiological validity of single-channel EEG acquisition from a mastoid electrode for emotion recognition. Despite the minimal setup, the recorded signals showed emotion-related modulations, confirming that the activity captured is of neural rather than artifactual origin. This result supports the use of simplified and wearable EEG configurations for affective monitoring in real-world contexts.

Statistical analyses revealed that absolute β- and γ-band power, together with entropy-based complexity measures (TD_SampEn, TD_PermEn), constitute the most informative and consistent EEG markers of emotional processing. The explainable AI analysis, based on the Gradient×Input method, confirmed and extended the statistical observations, providing interpretable relevance maps that quantitatively identified the dominant EEG features contributing to arousal and valence discrimination. The convergence between XAI-derived relevance and classical statistics strengthens the evidence that high-frequency oscillations and entropy measures jointly encode emotional intensity and valence. β power primarily reflect arousal-related engagement, while γ power is more sensitive to valence, particularly in contrasts involving neutral or emotionally charged states. Temporal complexity metrics complement these findings, indicating that nonlinear signal irregularity captures individualized aspects of emotional dynamics that go beyond spectral power changes.

Overall, these results demonstrate that meaningful and interpretable affective information can be extracted even from a single mastoid channel. The integration of rigorous pre-processing, individualized ANN–XAI modeling, and traditional statistical validation establishes a robust methodological framework for emotion decoding from minimal EEG configurations.

Future studies should extend this approach to larger and more heterogeneous populations to develop robust and generalizable models, explore domain adaptation strategies to mitigate inter-subject variability, and implement real-time emotion tracking to assess its applicability in ecological and wearable scenarios. Optimized solutions from an ergonomic perspective will be explored as well in order to confirm their feasibility under conditions where the sensing apparatus is minimized in size. In addition to this, future work will also investigate whether combining XAI-guided feature selection with more advanced classification architectures (e.g., temporal or graph-based models) can further enhance predictive performance while preserving interpretability.

## Figures and Tables

**Table 1:** Summary of EEG features showing statistically significant differences (*p* < 0.05, large effect size) in the intra-subject analysis for each participant. For both the arousal and valence dimensions, the table lists the features that exhibited significant contrasts across condition pairs (Low–High, Low–Mid, Mid–High). Entries marked as *n.s.* indicate non-significant comparisons.

Subject	Arousal			Valence		
	Low_vs_High	Low_vs_Mid	Mid_vs_High	Low_vs_High	Low_vs_Mid	Mid_vs_High
s01	HFD, Hjorth_Mobility, Hjorth C., LZC, PermEn, TD_PFD, TD_SampEn, TD_ZCR, $\beta_{2_{r}}$, $\beta_{3_{r}}$, $\beta_{r}$, $\gamma_{1_{r}}$, $\gamma_{2_{r}}$, $\gamma_{r}$, $\delta_{r}$	AGR, HFD, Hjorth_Mobility, Hjorth C., LZC, PermEn, TD_PFD, TD_SampEn, TD_ZCR, $\alpha_{1_{a}}$, $\alpha_{a}$, $\beta_{1_{r}}$, $\beta_{2_{r}}$, $\beta_{2_{a}}$, $\beta_{3_{r}}$, $\beta_{3_{a}}$, $\beta_{r}$, $\beta_{a}$, $\gamma_{1_{r}}$, $\gamma_{1_{a}}$, $\gamma_{2_{r}}$, $\gamma_{2_{a}}$, $\gamma_{r}$, $\gamma_{a}$, $\delta_{r}$	LZC, TD_ZCR, $\beta_{2_{r}}$, $\beta_{2_{a}}$, $\gamma_{2_{r}}$, $\delta_{r}$	n.s.	$\theta_{r}$	HFD, Hjorth_Mobility, Hjorth C., LZC, TD_SampEn, TD_ZCR, $\beta_{2_{r}}$, $\beta_{2_{a}}$, $\beta_{3_{r}}$, $\beta_{r}$, $\beta_{a}$, $\gamma_{2_{r}}$, $\gamma_{2_{a}}$, $\gamma_{r}$, $\delta_{r}$
s03	Hjorth A., $\gamma_{1_{a}}$, $\gamma_{2_{a}}$, $\gamma_{a}$, $\theta_{a}$	n.s.	Hjorth_Mobility, Hjorth A., LZC, PermEn, TD_PFD, TD_SampEn, TD_ZCR, $\alpha_{1_{a}}$, $\alpha_{2_{a}}$, $\alpha_{a}$, $\beta_{1_{a}}$, $\beta_{2_{a}}$, $\beta_{3_{r}}$, $\beta_{3_{a}}$, $\beta_{a}$, $\gamma_{1_{r}}$, $\gamma_{1_{a}}$, $\gamma_{2_{r}}$, $\gamma_{2_{a}}$, $\gamma_{r}$, $\gamma_{a}$, $\delta_{r}$	ABR	n.s.	HFD, $\beta_{2_{r}}$, $\beta_{2_{a}}$, $\beta_{3_{r}}$, $\beta_{3_{a}}$, $\beta_{r}$, $\beta_{a}$, $\gamma_{1_{r}}$, $\gamma_{1_{a}}$, $\gamma_{2_{r}}$, $\gamma_{2_{a}}$, $\gamma_{r}$, $\gamma_{a}$
s04	HFD, $\beta_{1_{r}}$, $\beta_{1_{a}}$, $\beta_{3_{r}}$, $\beta_{3_{a}}$, $\beta_{r}$, $\beta_{a}$, $\gamma_{1_{r}}$, $\gamma_{1_{a}}$, $\gamma_{2_{r}}$, $\gamma_{2_{a}}$, $\gamma_{r}$, $\gamma_{a}$	ABR, AGR, HFD, Hjorth_Mobility, Hjorth C., LZC, PermEn, TD_PFD, TD_SampEn, TD_ZCR, $\beta_{1_{r}}$, $\beta_{1_{a}}$, $\beta_{2_{r}}$, $\beta_{2_{a}}$, $\beta_{3_{r}}$, $\beta_{3_{a}}$, $\beta_{r}$, $\beta_{a}$, $\gamma_{1_{r}}$, $\gamma_{1_{a}}$, $\gamma_{2_{r}}$, $\gamma_{2_{a}}$, $\gamma_{r}$, $\gamma_{a}$, $\delta_{r}$	Hjorth C., $\beta_{2_{r}}$	TD_CV, $\alpha_{2_{a}}$, $\alpha_{a}$, $\beta_{1_{a}}$, $\beta_{2_{a}}$, $\beta_{3_{r}}$, $\beta_{3_{a}}$, $\beta_{a}$, $\gamma_{1_{a}}$, $\gamma_{2_{a}}$, $\gamma_{a}$	Hjorth_Mobility, Hjorth C., $\beta_{2_{r}}$	ABR, AGR, Hjorth_Mobility, Hjorth C., LZC, TD_SampEn, TD_ZCR, $\beta_{1_{a}}$, $\beta_{2_{r}}$, $\beta_{2_{a}}$, $\beta_{3_{r}}$, $\beta_{3_{a}}$, $\beta_{r}$, $\beta_{a}$, $\gamma_{1_{r}}$, $\gamma_{1_{a}}$, $\gamma_{2_{r}}$, $\gamma_{2_{a}}$, $\gamma_{r}$, $\gamma_{a}$, $\delta_{r}$
s05	n.s.	n.s.	n.s.	LZC, TD_SampEn, TD_ZCR, $\beta_{3_{a}}$, $\gamma_{1_{r}}$, $\gamma_{1_{a}}$, $\gamma_{a}$	LZC, TD_ZCR, $\beta_{2_{a}}$, $\beta_{a}$, $\gamma_{1_{a}}$, $\gamma_{a}$	n.s.
s06	n.s.	n.s.	n.s.	ABR, AGR, HFD, Hjorth_Mobility, Hjorth C., LZC, PermEn, TD_CV, TD_PFD, TD_SampEn, TD_Skewness, TD_ZCR, $\alpha_{1_{r}}$, $\alpha_{r}$, $\beta_{1_{r}}$, $\beta_{1_{a}}$, $\beta_{2_{r}}$, $\beta_{2_{a}}$, $\beta_{3_{r}}$, $\beta_{3_{a}}$, $\beta_{r}$, $\beta_{a}$, $\gamma_{1_{r}}$, $\gamma_{1_{a}}$, $\gamma_{2_{r}}$, $\gamma_{2_{a}}$, $\gamma_{r}$, $\gamma_{a}$, $\delta_{r}$, $\theta_{r}$	ABR, AGR, HFD, Hjorth_Mobility, Hjorth A., Hjorth C., LZC, PermEn, TD_PFD, TD_SampEn, TD_ZCR, $\alpha_{1_{r}}$, $\alpha_{r}$, $\beta_{2_{r}}$, $\beta_{2_{a}}$, $\beta_{3_{r}}$, $\beta_{3_{a}}$, $\beta_{r}$, $\beta_{a}$, $\gamma_{1_{r}}$, $\gamma_{1_{a}}$, $\gamma_{2_{r}}$, $\gamma_{2_{a}}$, $\gamma_{r}$, $\gamma_{a}$, $\theta_{r}$	ABR, Hjorth_Mobility, Hjorth C., LZC, TD_CV, TD_SampEn, TD_ZCR, $\beta_{3_{r}}$, $\delta_{r}$
s07	HFD, Hjorth C., LZC, TD_PFD, $\beta_{3_{a}}$, $\gamma_{1_{a}}$, $\gamma_{2_{a}}$, $\gamma_{a}$	Hjorth_Mobility, Hjorth A., TD_ZCR, $\beta_{2_{a}}$, $\gamma_{1_{a}}$, $\gamma_{2_{a}}$, $\gamma_{a}$	TD_SampEn, $\beta_{3_{a}}$, $\gamma_{1_{a}}$	$\beta_{2_{a}}$, $\beta_{3_{a}}$, $\gamma_{1_{a}}$	Hjorth A., LZC, TD_ZCR, $\beta_{2_{a}}$, $\gamma_{a}$	TD_PFD, $\beta_{2_{a}}$, $\beta_{3_{a}}$, $\gamma_{a}$
s08	Hjorth C., LZC, TD_ZCR, $\beta_{3_{a}}$, $\gamma_{a}$	$\beta_{2_{a}}$, $\gamma_{a}$	HFD, LZC, TD_PFD, TD_ZCR, $\beta_{3_{a}}$, $\gamma_{a}$	HFD, Hjorth C., TD_PFD, TD_ZCR, $\beta_{3_{a}}$, $\gamma_{a}$	Hjorth A., TD_ZCR, $\beta_{2_{a}}$	LZC, TD_SampEn, $\beta_{3_{a}}$
s09	HFD, Hjorth_Mobility, TD_ZCR, $\beta_{2_{a}}$, $\beta_{3_{a}}$, $\gamma_{a}$	LZC, TD_ZCR, $\beta_{3_{a}}$	TD_SampEn, TD_ZCR, $\beta_{3_{a}}$, $\gamma_{a}$	Hjorth C., TD_SampEn, $\beta_{3_{a}}$	Hjorth A., TD_ZCR, $\beta_{2_{a}}$, $\gamma_{a}$	LZC, TD_ZCR, $\beta_{3_{a}}$, $\gamma_{a}$
s10	HFD, Hjorth C., LZC, TD_PFD, TD_ZCR, $\beta_{3_{a}}$, $\gamma_{a}$	TD_ZCR, $\beta_{3_{a}}$	Hjorth_Mobility, TD_SampEn, $\beta_{3_{a}}$, $\gamma_{a}$	Hjorth A., LZC, TD_ZCR, $\beta_{3_{a}}$	Hjorth C., TD_SampEn, $\beta_{3_{a}}$, $\gamma_{a}$	TD_ZCR, $\beta_{3_{a}}$
s11	Hjorth C., TD_SampEn, TD_ZCR, $\beta_{3_{a}}$, $\gamma_{a}$	HFD, TD_ZCR, $\beta_{3_{a}}$	Hjorth_Mobility, Hjorth A., TD_ZCR, $\beta_{3_{a}}$	Hjorth C., TD_SampEn, $\beta_{3_{a}}$, $\gamma_{a}$	LZC, TD_PFD, TD_ZCR, $\beta_{3_{a}}$	Hjorth_Mobility, TD_ZCR, $\beta_{3_{a}}$
s12	HFD, Hjorth_Mobility, TD_PFD, TD_ZCR, $\beta_{3_{a}}$, $\gamma_{a}$	Hjorth C., TD_ZCR, $\beta_{3_{a}}$	TD_ZCR, $\beta_{3_{a}}$, $\gamma_{a}$	Hjorth A., TD_SampEn, TD_ZCR, $\beta_{3_{a}}$	TD_ZCR, $\beta_{3_{a}}$, $\gamma_{a}$	Hjorth C., TD_ZCR, $\beta_{3_{a}}$
s13	HFD, Hjorth C., TD_SampEn, TD_ZCR, $\beta_{3_{a}}$	Hjorth_Mobility, TD_ZCR, $\beta_{3_{a}}$	TD_PFD, $\beta_{3_{a}}$, $\gamma_{a}$	TD_ZCR, $\beta_{3_{a}}$, $\gamma_{a}$	Hjorth C., TD_SampEn, TD_ZCR, $\beta_{3_{a}}$	Hjorth_Mobility, TD_ZCR, $\beta_{3_{a}}$
s14	Hjorth A., TD_SampEn, TD_ZCR, $\beta_{3_{a}}$, $\gamma_{a}$	HFD, Hjorth C., TD_ZCR, $\beta_{3_{a}}$	TD_SampEn, TD_ZCR, $\beta_{3_{a}}$	Hjorth_Mobility, TD_ZCR, $\beta_{3_{a}}$	TD_ZCR, $\beta_{3_{a}}$	Hjorth A., TD_SampEn, TD_ZCR, $\beta_{3_{a}}$
s15	Hjorth_Mobility, TD_PFD, TD_ZCR, $\beta_{3_{a}}$	TD_ZCR, $\beta_{3_{a}}$, $\gamma_{a}$	HFD, TD_SampEn, TD_ZCR, $\beta_{3_{a}}$	Hjorth C., TD_ZCR, $\beta_{3_{a}}$, $\gamma_{a}$	TD_ZCR, $\beta_{3_{a}}$	Hjorth_Mobility, TD_ZCR, $\beta_{3_{a}}$
s16	Hjorth A., TD_ZCR, $\beta_{3_{a}}$, $\gamma_{a}$	HFD, Hjorth C., TD_PFD, TD_SampEn, TD_ZCR, $\beta_{3_{a}}$	TD_ZCR, $\beta_{3_{a}}$	Hjorth_Mobility, TD_ZCR, $\beta_{3_{a}}$	TD_ZCR, $\beta_{3_{a}}$	Hjorth C., TD_ZCR, $\beta_{3_{a}}$
s17	Hjorth C., TD_SampEn, TD_ZCR, $\beta_{3_{a}}$	TD_ZCR, $\beta_{3_{a}}$, $\gamma_{a}$	Hjorth_Mobility, TD_ZCR, $\beta_{3_{a}}$	HFD, Hjorth C., TD_ZCR, $\beta_{3_{a}}$	TD_ZCR, $\beta_{3_{a}}$, $\gamma_{a}$	Hjorth_Mobility, TD_ZCR, $\beta_{3_{a}}$
s18	Hjorth C., TD_SampEn, TD_ZCR, $\beta_{3_{a}}$, $\gamma_{a}$	HFD, TD_ZCR, $\beta_{3_{a}}$	Hjorth_Mobility, TD_ZCR, $\beta_{3_{a}}$	TD_ZCR, $\beta_{3_{a}}$	Hjorth C., TD_SampEn, TD_ZCR, $\beta_{3_{a}}$	Hjorth_Mobility, TD_ZCR, $\beta_{3_{a}}$
s19	Hjorth_Mobility, TD_SampEn, TD_ZCR, $\beta_{3_{a}}$	TD_ZCR, $\beta_{3_{a}}$, $\gamma_{a}$	HFD, Hjorth C., TD_PFD, TD_ZCR, $\beta_{3_{a}}$	Hjorth_Mobility, TD_ZCR, $\beta_{3_{a}}$	TD_ZCR, $\beta_{3_{a}}$, $\gamma_{a}$	Hjorth C., TD_ZCR, $\beta_{3_{a}}$
s20	Hjorth C., TD_SampEn, TD_ZCR, $\beta_{3_{a}}$, $\gamma_{a}$	HFD, TD_ZCR, $\beta_{3_{a}}$	Hjorth_Mobility, TD_ZCR, $\beta_{3_{a}}$	TD_ZCR, $\beta_{3_{a}}$	Hjorth C., TD_SampEn, TD_ZCR, $\beta_{3_{a}}$	Hjorth_Mobility, TD_ZCR, $\beta_{3_{a}}$
s21	Hjorth A., TD_ZCR, $\beta_{3_{a}}$, $\gamma_{a}$	Hjorth C., TD_SampEn, TD_ZCR, $\beta_{3_{a}}$	TD_ZCR, $\beta_{3_{a}}$	HFD, Hjorth C., TD_ZCR, $\beta_{3_{a}}$	TD_ZCR, $\beta_{3_{a}}$, $\gamma_{a}$	Hjorth_Mobility, TD_ZCR, $\beta_{3_{a}}$
s22	Hjorth_Mobility, TD_SampEn, TD_ZCR, $\beta_{3_{a}}$	TD_ZCR, $\beta_{3_{a}}$, $\gamma_{a}$	Hjorth C., TD_ZCR, $\beta_{3_{a}}$	Hjorth_Mobility, TD_ZCR, $\beta_{3_{a}}$	TD_ZCR, $\beta_{3_{a}}$	HFD, Hjorth C., TD_ZCR, $\beta_{3_{a}}$
s23	Hjorth C., TD_SampEn, TD_ZCR, $\beta_{3_{a}}$, $\gamma_{a}$	HFD, TD_ZCR, $\beta_{3_{a}}$	Hjorth_Mobility, TD_ZCR, $\beta_{3_{a}}$	TD_ZCR, $\beta_{3_{a}}$	Hjorth C., TD_SampEn, TD_ZCR, $\beta_{3_{a}}$	Hjorth_Mobility, TD_ZCR, $\beta_{3_{a}}$
s24	Hjorth A., TD_ZCR, $\beta_{3_{a}}$, $\gamma_{a}$	Hjorth C., TD_SampEn, TD_ZCR, $\beta_{3_{a}}$	TD_ZCR, $\beta_{3_{a}}$	HFD, Hjorth C., TD_ZCR, $\beta_{3_{a}}$	TD_ZCR, $\beta_{3_{a}}$, $\gamma_{a}$	Hjorth_Mobility, TD_ZCR, $\beta_{3_{a}}$

**Table 2:** Summary of EEG features that reached statistical significance (p<0.05) and exhibited at least a large effect size identified in the across-subjects analysis. The table reports for both arousal and valence conditions the consistency of the feature, the normalized effect size, the median *p*-value, and the corresponding score.

Condition	Comparison	Features	Consistency	Normalized Effect Size	Median *p*-Value	Score
Arousal	mid_vs_high	$\beta_{a}$	86%	1.63	8.93E-49	16.80
	mid_vs_high	$\beta_{a_{1}}$	86%	1.60	1.45E-50	16.43
	mid_vs_high	$\gamma_{a}$	75%	1.55	4.10E-52	13.94
	mid_vs_high	$\gamma_{a_{2}}$	75%	1.53	2.52E-42	13.79
	mid_vs_high	$\gamma_{a_{1}}$	75%	1.53	7.59E-45	13.73
	mid_vs_high	$\beta_{a_{2}}$	75%	1.49	2.33E-36	13.37
	mid_vs_high	$\beta_{a_{3}}$	71%	1.52	3.68E-38	13.04
	mid_vs_high	Hjorth_Activity	75%	1.44	3.57E-38	13.00
	low_vs_mid	$\gamma_{a}$	57%	1.40	7.52E-52	9.63
	low_vs_mid	$\gamma_{a_{2}}$	57%	1.39	5.04E-42	9.50
	low_vs_mid	$\beta_{a}$	50%	1.32	2.24E-45	7.91
Valence	mid_vs_high	Complexity_Index	71%	1.63	5.68E-26	13.97
	low_vs_high	$\beta_{a_{3}}$	71%	1.55	4.91E-55	13.33
	mid_vs_high	Hjorth_Mobility	71%	1.55	2.85E-23	13.28
	low_vs_high	$\gamma_{a_{2}}$	71%	1.49	1.22E-37	12.74
	low_vs_high	$\gamma_{a_{1}}$	71%	1.48	1.53E-48	12.65
	mid_vs_high	$\beta_{a_{2}}$	71%	1.45	3.85E-29	12.42
	low_vs_high	Arousal_Index	71%	1.42	7.47E-26	12.20
	mid_vs_high	$\gamma_{a_{2}}$	71%	1.42	1.06E-25	12.17
	mid_vs_high	$\beta_{a}$	71%	1.37	2.72E-30	11.75
	low_vs_high	$\gamma_{a}$	63%	1.43	3.46E-37	10.70
	low_vs_high	$\beta_{a_{2}}$	57%	1.40	1.68E-45	9.57
	low_vs_mid	$\gamma_{a}$	57%	1.36	6.92E-37	9.36
	low_vs_high	AGR	57%	1.30	6.22E-23	8.90
	low_vs_high	$\beta_{a}$	56%	1.32	1.17E-40	8.82
	mid_vs_high	$\beta_{a_{1}}$	57%	1.23	3.92E-27	8.44
	low_vs_high	$\beta_{a_{1}}$	57%	1.19	1.59E-24	8.13
	low_vs_mid	$\beta_{a}$	50%	1.32	1.08E-28	7.89
	low_vs_mid	$\gamma_{a_{2}}$	50%	1.31	5.22E-26	7.85

**Table 3:** Valence classification task: intra-subject cumulative relevance scores obtained through the Gradient × Input analysis. Each value represents the summed contribution of a given EEG feature across all subjects in which it was identified as relevant (normalized relevance > 0.5). Columns correspond to spectral, statistical, and complexity-related features. The “Total” row reports the aggregate contribution across both low- and high-valence classes. ‘Hjorth C.’ = Hjorth Complexity; ‘Hjorth M.’ = Hjorth Mobility.

Class	TD PermEn	TD PFD	$\beta_{1_{a}}$	$\alpha_{2_{r}}$	$\beta_{3_{a}}$	TD ZCR	$\theta_{r}$	Hjorth C.	Hjorth M.	$\beta_{3_{r}}$	TD HFD	$\gamma_{2_{r}}$	$\alpha_{2_{a}}$	$\beta_{2_{r}}$	TD LZC	TD SampEn
Low	2.102	1.304	2.114	0.965	0.537	1.304	1.798	2.770	2.687	1.545	3.294	3.016	2.701	3.837	3.676	3.846
High	1.803	1.023	1.671	1.056	0.525	1.626	2.353	2.730	2.940	1.349	3.287	2.944	2.703	3.745	3.956	3.663
Total	3.905	2.327	3.785	2.021	1.062	2.930	4.151	5.499	5.628	2.894	6.581	5.960	5.405	7.582	7.632	7.509

**Table 4:** Arousal classification task: intra-subject cumulative relevance scores obtained through the Gradient × Input analysis. Each value represents the summed contribution of a given EEG feature across all subjects in which it was identified as relevant (normalized relevance > 0.5). Columns correspond to spectral, statistical, and complexity-related features. The “Total” row reports the aggregate contribution across both low- and high-arousal classes. ‘Hjorth C.’ = Hjorth Complexity.

Class	ABR	AGR	$\beta_{2_{a}}$	$\beta_{3_{a}}$	$\delta_{a}$	$\gamma_{1_{a}}$	$\gamma_{2_{a}}$	$\theta_{a}$	Hjorth C.	$\alpha_{1_{r}}$	$\alpha_{2_{r}}$
Low	2.271	0.994	1.526	1.180	0.545	0.951	2.265	0.581	2.184	0.926	1.741
High	1.950	1.125	1.474	1.007	0.730	1.112	2.579	0.644	2.314	1.015	1.850
Total	4.221	2.119	3.000	2.186	1.275	2.063	4.845	1.225	4.498	1.941	3.591
	$\beta_{1_{r}}$	$\beta_{2_{r}}$	$\gamma_{1_{r}}$	$\gamma_{2_{r}}$	$\theta_{r}$	**TD HFD**	**TD Kurtosis**	**TD LZC**	**TD PFD**	**TD PermEn**	**TD SampEn**
Low	0.906	1.847	0.795	2.966	2.371	4.190	1.213	3.289	0.905	0.919	3.722
High	0.820	1.686	0.896	3.004	2.908	4.292	1.055	3.447	0.753	0.904	3.378
Total	1.726	3.533	1.691	5.970	5.278	8.482	2.268	6.736	1.658	1.824	7.099

**Table 5:** Valence classification task: intra-subject cumulative relevance scores obtained through the Integrated Gradient analysis. Each value represents the summed contribution of a given EEG feature across all subjects in which it was identified as relevant (normalized relevance > 0.5). Columns correspond to spectral, statistical, and complexity-related features. The “Total” row reports the aggregate contribution across both low- and high-valence classes. ‘Hjorth C.’ = Hjorth Complexity.

Class	ABR	$\beta_{2_{a}}$	$\delta_{a}$	Hjorth C.	TD PermEn	TD PFD	$\beta_{1_{r}}$	$\beta_{3_{a}}$	TD LZC	TD SampEn	TD HFD	$\theta_{r}$
Low	2.756	3.121	2.215	3.129	3.415	3.647	3.264	3.623	4.570	3.927	5.475	5.398
High	2.992	2.375	3.064	2.757	3.174	3.172	2.664	2.941	4.231	4.740	6.144	5.971
Total	5.748	5.497	5.279	5.886	6.589	6.819	5.928	6.565	8.800	8.667	11.619	11.370

**Table 6:** Arousal classification task: intra-subject cumulative relevance scores obtained through the Integrated Gradient analysis. Each value represents the summed contribution of a given EEG feature across all subjects in which it was identified as relevant (normalized relevance > 0.5). Columns correspond to spectral, statistical, and complexity-related features. The “Total” row reports the aggregate contribution across both low- and high-arousal classes. ‘Hjorth C.’ = Hjorth Complexity.

Class	ABR	AGR	$\theta_{r}$	TD HFD	$\beta_{a}$	$\alpha_{1_{a}}$	$\beta_{3_{a}}$	TD ZCR	Hjorth C.	$\alpha_{1_{r}}$	$\beta_{2_{r}}$	$\gamma_{r}$
Low	1.050	1.912	1.466	1.537	1.051	1.441	1.662	1.386	2.128	2.051	1.908	2.596
High	1.635	1.628	1.280	1.634	0.691	1.309	1.407	1.478	1.618	2.334	1.475	1.929
Total	2.685	3.540	2.746	3.171	1.742	2.750	3.069	2.864	3.746	4.385	3.383	4.525
	**TD** **PermEn**	**TD** **PFD**	$\gamma_{2_{r}}$	$\delta_{r}$	$\theta_{a}$	$\gamma_{1_{a}}$	$\gamma_{a}$	$\beta_{3_{r}}$	$\beta_{1_{a}}$	$\gamma_{2_{a}}$	**TD** **LZC**	**TD** **SampEn**
Low	1.944	1.626	2.843	1.993	2.403	2.399	3.012	3.874	3.214	3.474	4.219	4.604
High	1.418	1.215	3.199	1.483	2.481	1.629	2.778	1.818	4.007	4.917	3.712	4.148
Total	3.362	2.841	6.041	3.476	4.884	4.027	5.790	5.692	7.221	8.391	7.931	8.752

## Data Availability

The raw data supporting the conclusions of this article will be made available by the authors on request.

## Abbreviations

The following abbreviations are used in this manuscript:

EEG	Electroencephalography
PSD	Power Spectral Density
ECG	Electrocardiogram
GSR	Galvanic Skin Respiration
RR	Respiration Rate
ST	Skin Temperature
CNNs	Convolutional Neural Networks
SVM	Support Vector Machine
CFNN	Convolutional Fuzzy Neural Network
XAI	eXplainable Artificial Intelligence
LV	Low valence
HA	High arousal
LA	Low arousal
HV	High valence
QC	Quality Control
HF	High Frequency
LF	Low Frequency
SAM	Self Assessment Manikin
ASP	Absolute Spectral Power
RSP	Relative Spectral Power
ABR	Alpha–Beta Ratio
AGR	Alpha–Gamma Ratio
ZCR	Zero-Crossing Rate
PFD	Petrosian Fractal Dimensions
HFD	Higuchi Fractal Dimensions
LZC	Lempel–Ziv Complexity
PermEn	Permutation Entropy
SampEn	Sample Entropy
CV	Coefficient of Variation
FDR_BH_	Benjamini–Hochberg False Discovery Rate
ANN	Artificial Neural Network
LOSO	Leave-One-Subject-Out
kNN	K-Nearest Neighbors
LR	Logistic Regression
GI	Integrated Gradient

## Appendix A

**Table A1:** Summary of the 39 EEG features extracted from the single-channel ear-EEG signal, grouped into spectral/energy-related, statistical/temporal, and complexity-based descriptors.

Category of Feature	Subcategory	Features
Spectral/energy-related	Absolute Power	$\delta_{a}$, $\theta_{a}$, $\alpha_{a}$, $\alpha_{1_{a}}$, $\alpha_{2_{a}}$, $\beta_{a}$, $\beta_{1_{a}}$, $\beta_{2_{a}}$, $\beta_{3_{a}}$, $\gamma_{a}$, $\gamma_{1_{a}}$, $\gamma_{2_{a}}$
	Relative Power	$\delta_{r}$, $\theta_{r}$, $\alpha_{r}$, $\alpha_{1_{r}}$, $\alpha_{2_{r}}$, $\beta_{r}$, $\beta_{1_{r}}$, $\beta_{2_{r}}$, $\beta_{3_{r}}$, $\gamma_{r}$, $\gamma_{1_{r}}$, $\gamma_{2_{r}}$, *Total Power*
	Spectral Ratios	ABR, AGR
Statistical/temporal	–	TD_Skewness, TD_Kurtosis, TD_CV
Complexity measures	–	TD_HFD, TD_LZC, TD_PFD, TD_PermEn, TD_SampEn
	–	TD_ZCR, Hjorth_Activity, Hjorth_Complexity, Hjorth_Mobility

**Table A2:** QC scores registered for the excluded videos.

Subject	Video	Max Abs Score	Max Jump Score	Peak-to-Peak Score	HF Ratio Score	LF Ratio Score	Epoch Retention
s02	Neutral_6	100	12	100	22	98	98
s02	EQ_2	100	33	100	15	98	98
s02	EC_3	100	96	100	31	99	98
s06	Scenery_1	100	52	100	5	98	99
s06	Erotic_6	100	3	100	13	99	98
s06	Erotic_4	100	7	100	15	99	98
s07	Neutral_6	100	60	100	46	98	97
s07	EQ_1	100	100	100	44	92	94
s07	EQ_2	100	100	100	46	99	97
s07	Neutral_4	100	9	100	11	98	96
s08	Neutral_6	100	45	100	15	96	95
s08	Sadness_3	100	40	100	26	92	95
s08	EQ_2	100	40	100	32	97	94
s08	Neutral_2	100	50	100	29	95	94
s08	EC_3	100	100	100	19	95	94
s08	EQ_1	100	97	100	40	94	93
s08	EQ_2	100	57	100	33	95	93
s08	Neutral_6	100	100	100	55	94	92
s08	Neutral_4	100	100	100	65	96	92
s08	EQ_1	100	100	100	65	95	96
s14	Scenery_2	100	37	100	32	90	91
s22	EQ_1	100	100	100	33	93	93
s02	EQ_2	100	100	100	40	100	100
s02	EC_3	100	40	100	29	92	92
s02	EQ_2	100	100	100	47	99	100
s24	Relaxed_7	100	40	100	40	93	94
s24	Relaxed_4	100	100	100	45	99	100
s24	Relaxed_3	100	60	100	50	92	100

**Table A3:** Intra-subject optimal ANN configurations and corresponding arousal classification performance. For each subject, the model architecture (number of hidden layers, nodes per layer, activation function, batch size, and weight decay) yielding the highest test accuracy (mean ± std) is reported.

Subject	N. Layers	Nodes List	Activation	Batch Size	Weight Decay	Test Acc (Mean ± Std)
s03	1	[16]	tanh	256	0	68.21 ± 15.91%
s04	1	[32]	relu	256	0	76.72 ± 18.98%
s05	1	[32]	tanh	256	0	56.63 ± 17.27%
s06	3	[64, 32, 16]	tanh	256	0	64.12 ± 20.58%
s08	3	[64, 32, 16]	tanh	256	0	73.49 ± 17.41%
s09	1	[128]	tanh	128	0	80.93 ± 22.63%
s10	1	[128]	relu	128	0	78.14 ± 15.49%
s11	1	[128]	tanh	128	0	75.24 ± 16.78%
s12	2	[128, 64]	tanh	128	0	67.02 ± 15.76%
s14	1	[32]	relu	128	0	69.63 ± 17.54%
s17	1	[64]	tanh	256	0	71.26 ± 18.10%
s19	3	[64, 32, 16]	tanh	256	0	61.51 ± 7.91%
s20	1	[64]	tanh	256	0	75.94 ± 8.19%
s21	3	[128, 32, 16]	tanh	128	0	73.95 ± 12.56%
s23	2	[64, 32]	tanh	256	0	76.05 ± 18.81%
s24	3	[128, 64, 16]	relu	128	0	80.03 ± 14.84%

**Table A4:** Intra-Subject optimal ANN configurations and corresponding valence classification performance. For each subject, the model architecture (number of hidden layers, nodes per layer, activation function, batch size, and weight decay) yielding the highest test accuracy (mean ± std) is reported.

Subject	N. Layers	Nodes List	Activation	Batch Size	Weight Decay	Test Acc (Mean ± Std)
s03	3	[64, 32, 16]	tanh	256	0	75.54 ± 15.05%
s04	1	[128]	tanh	128	0	58.13 ± 15.42%
s05	1	[128]	relu	128	0	85.05 ± 10.28%
s06	1	[128]	tanh	128	0	95.90 ± 5.09%
s08	2	[128, 64]	tanh	128	0	74.35 ± 16.54%
s09	1	[32]	relu	128	0	78.90 ± 17.88%
s10	1	[64]	tanh	256	0	61.18 ± 17.22%
s11	3	[64, 32, 16]	tanh	256	0	72.39 ± 12.15%
s12	1	[64]	tanh	256	0	63.49 ± 14.71%
s14	3	[128, 32, 16]	tanh	128	0	66.76 ± 6.78%
s17	2	[64, 32]	tanh	256	0	67.21 ± 12.53%
s19	3	[128, 64, 16]	relu	128	0	60.58 ± 18.34%
s20	1	[16]	tanh	256	0	50.82 ± 17.69%
s21	1	[32]	relu	256	0	62.96 ± 17.60%
s23	1	[32]	tanh	256	0	60.19 ± 16.39%
s24	3	[64, 32, 16]	tanh	256	0	90.51 ± 11.01%

**Table A5:** Valence classification performance for each subject. For each subject, training and test accuracies, precision, recall, and F1 score (mean ± std) are reported, along with the corresponding performance of SVM, KNN, and LR classifiers. Metrics are computed based on the optimal ANN configuration for each individual.

Subject	Train Acc (%) Mean ± Std	Test Acc (%) Mean ± Std	Precision (%) Mean ± Std	Recall (%) Mean ± Std	F1 (%) Mean ± Std	SVM (%) Mean ± Std	KNN (%) Mean ± Std	LR (%) Mean ± Std
s03	100.00 ± 0.00	75.54 ± 15.05	76.32 ± 15.28	75.54 ± 15.05	75.36 ± 15.13	73.16 ± 22.48	72.29 ± 14.69	64.89 ± 11.07
s04	100.00 ± 0.00	58.13 ± 15.42	59.07 ± 16.64	58.13 ± 15.42	57.45 ± 15.49	46.17 ± 11.52	49.82 ± 5.55	45.89 ± 14.09
s05	100.00 ± 0.00	85.05 ± 10.28	86.32 ± 9.95	85.05 ± 10.28	84.85 ± 10.46	59.79 ± 8.86	57.36 ± 6.73	56.15 ± 10.86
s06	99.96 ± 0.12	95.90 ± 5.09	96.49 ± 3.82	95.90 ± 5.09	95.84 ± 5.25	84.88 ± 13.26	83.33 ± 10.12	80.84 ± 14.24
s08	99.88 ± 0.27	74.35 ± 16.54	79.28 ± 13.64	74.35 ± 16.54	71.43 ± 20.06	61.77 ± 19.40	68.54 ± 16.80	56.35 ± 16.94
s09	100.00 ± 0.00	78.90 ± 17.88	78.65 ± 23.58	78.90 ± 17.88	76.78 ± 21.42	65.47 ± 14.09	60.60 ± 15.38	61.39 ± 11.47
s10	100.00 ± 0.00	61.18 ± 17.22	61.39 ± 18.13	61.18 ± 17.22	60.14 ± 18.04	61.84 ± 16.88	51.85 ± 15.72	54.86 ± 16.91
s11	99.92 ± 0.17	72.39 ± 12.15	74.52 ± 12.70	72.39 ± 12.15	71.78 ± 12.38	62.24 ± 10.67	63.93 ± 10.59	64.49 ± 13.95
s12	99.84 ± 0.50	63.49 ± 14.71	64.29 ± 15.10	63.49 ± 14.71	62.73 ± 15.14	52.29 ± 15.63	50.20 ± 9.75	42.53 ± 13.09
s14	99.93 ± 0.21	66.76 ± 6.78	68.16 ± 7.66	66.76 ± 6.78	66.19 ± 6.80	58.62 ± 12.09	59.16 ± 5.55	55.77 ± 16.74
s17	100.00 ± 0.00	67.21 ± 12.53	68.64 ± 14.45	67.21 ± 12.53	65.52 ± 14.56	54.72 ± 10.61	61.69 ± 11.61	54.62 ± 14.34
s19	99.37 ± 0.88	60.58 ± 18.34	62.97 ± 21.02	60.58 ± 18.34	57.69 ± 20.06	52.48 ± 16.52	52.48 ± 12.70	52.33 ± 16.97
s20	99.43 ± 0.60	50.82 ± 17.69	50.66 ± 17.92	50.82 ± 17.69	50.42 ± 17.87	44.71 ± 13.74	43.25 ± 13.87	44.87 ± 7.63
s21	99.91 ± 0.09	62.96 ± 17.60	63.53 ± 17.54	62.96 ± 17.60	61.97 ± 18.20	54.44 ± 11.16	52.04 ± 8.68	49.45 ± 11.71
s23	99.98 ± 0.07	60.19 ± 16.39	60.58 ± 20.16	60.19 ± 16.39	57.75 ± 18.42	49.53 ± 8.89	46.55 ± 10.43	35.78 ± 14.22
s24	99.08 ± 0.55	90.51 ± 11.01	92.49 ± 7.87	90.51 ± 11.01	90.02 ± 12.04	53.10 ± 21.35	48.90 ± 17.31	61.31 ± 20.07

**Table A6:** Arousal classification performance for each subject. For each subject, training and test accuracies, precision, recall, and F1 score (mean ± std) are reported, along with the corresponding performance of SVM, KNN, and LR classifiers. Metrics are computed based on the optimal ANN configuration for each individual.

Subject	Train Acc (%) Mean ± Std	Test Acc (%) Mean ± Std	Precision (%) Mean ± Std	Recall (%) Mean ± Std	F1 (%) Mean ± Std	SVM (%) Mean ± Std	KNN (%) Mean ± Std	LR (%) Mean ± Std
s03	99.52 ± 0.58	68.21 ± 15.91	72.55 ± 16.16	68.21 ± 15.91	65.45 ± 17.84	67.70 ± 11.03	63.96 ± 12.15	67.18 ± 7.67
s04	100.00 ± 0.00	76.72 ± 18.98	79.72 ± 17.70	76.72 ± 18.98	75.36 ± 20.62	38.36 ± 12.79	44.68 ± 11.70	30.72 ± 13.73
s05	99.70 ± 0.56	56.63 ± 17.27	57.93 ± 20.26	56.63 ± 17.27	54.93 ± 17.96	39.56 ± 9.39	42.87 ± 7.53	35.19 ± 11.48
s06	99.84 ± 0.50	64.12 ± 20.58	65.81 ± 21.80	64.12 ± 20.58	63.31 ± 20.82	60.17 ± 16.90	62.68 ± 16.44	51.58 ± 18.09
s08	100.00 ± 0.00	73.49 ± 17.41	77.18 ± 17.63	73.49 ± 17.41	71.99 ± 18.66	57.42 ± 16.63	58.21 ± 14.81	47.90 ± 11.03
s09	100.00 ± 0.00	80.93 ± 22.63	81.88 ± 22.83	80.93 ± 22.63	80.50 ± 23.04	64.51 ± 10.15	67.91 ± 11.30	49.47 ± 14.48
s10	99.73 ± 0.83	78.14 ± 15.49	81.09 ± 14.90	78.14 ± 15.49	77.26 ± 16.17	63.09 ± 16.87	59.08 ± 20.68	61.16 ± 17.17
s11	100.00 ± 0.00	75.24 ± 16.78	76.41 ± 17.02	75.24 ± 16.78	74.82 ± 17.05	59.60 ± 10.62	50.02 ± 9.95	54.96 ± 14.49
s12	99.65 ± 0.62	67.02 ± 15.76	67.49 ± 16.73	67.02 ± 15.76	66.28 ± 16.64	47.12 ± 9.95	36.83 ± 7.43	41.69 ± 9.37
s14	99.65 ± 0.50	69.63 ± 17.54	69.98 ± 17.79	69.63 ± 17.54	69.34 ± 17.81	44.96 ± 9.47	47.73 ± 7.63	38.80 ± 11.84
s17	100.00 ± 0.00	71.26 ± 18.10	75.99 ± 18.19	71.26 ± 18.10	69.15 ± 19.68	56.62 ± 13.69	57.63 ± 9.54	45.26 ± 15.77
s19	98.43 ± 1.30	61.51 ± 7.91	61.92 ± 7.94	61.51 ± 7.91	61.17 ± 8.01	42.40 ± 12.65	46.12 ± 10.91	40.67 ± 12.64
s20	99.81 ± 0.59	75.94 ± 8.19	79.42 ± 8.73	75.94 ± 8.19	75.14 ± 8.67	60.96 ± 13.18	60.18 ± 13.56	56.42 ± 11.79
s21	99.79 ± 0.27	73.95 ± 12.56	75.45 ± 13.30	73.95 ± 12.56	73.66 ± 12.56	50.93 ± 12.48	53.04 ± 5.86	43.21 ± 13.53
s23	100.00 ± 0.00	76.05 ± 18.81	79.92 ± 18.66	76.05 ± 18.81	75.01 ± 19.45	54.85 ± 18.06	53.30 ± 18.75	60.26 ± 19.00
s24	100.00 ± 0.00	80.03 ± 14.84	79.84 ± 20.87	80.03 ± 14.84	77.85 ± 18.80	63.35 ± 14.43	66.05 ± 7.73	64.21 ± 15.22
